# Distribution of *Angiostrongylus vasorum* and its gastropod intermediate hosts along the rural–urban gradient in two cities in the United Kingdom, using real time PCR

**DOI:** 10.1186/s13071-016-1338-3

**Published:** 2016-02-02

**Authors:** Nor Azlina A. Aziz, Elizabeth Daly, Simon Allen, Ben Rowson, Carolyn Greig, Dan Forman, Eric R. Morgan

**Affiliations:** University of Bristol, Veterinary Parasitology & Ecology Group, School of Biological Sciences, Bristol Life Sciences Building, 24, Tyndall Avenue, Bristol, BS8 1TQ UK; School of Animal Sciences, Faculty of Bioresources and Food Industry, Universiti Sultan Zainal Abidin, Kampus Tembila, 22200 Terengganu, Malaysia; Department of Biosciences, Swansea University, Singleton Park, Swansea, SA2 8PP UK; Department of Natural Sciences, National Museum of Wales, Cardiff, Wales CF10 3NP UK

**Keywords:** Angiostrongylosis, Suburban, Gastropods, Epidemiology, Distribution

## Abstract

**Background:**

*Angiostrongylus vasorum* is a highly pathogenic metastrongylid nematode affecting dogs, which uses gastropod molluscs as intermediate hosts. The geographical distribution of the parasite appears to be heterogeneous or patchy and understanding of the factors underlying this heterogeneity is limited. In this study, we compared the species of gastropod present and the prevalence of *A. vasorum* along a rural–urban gradient in two cities in the south-west United Kingdom.

**Methods:**

The study was conducted in Swansea in south Wales (a known endemic hotspot for *A. vasorum*) and Bristol in south-west England (where reported cases are rare). In each location, slugs were sampled from nine sites across three broad habitat types (urban, suburban and rural). A total of 180 slugs were collected in Swansea in autumn 2012 and 338 in Bristol in summer 2014. A 10 mg sample of foot tissue was tested for the presence of *A. vasorum* by amplification of the second internal transcribed spacer (ITS-2) using a previously validated real-time PCR assay.

**Results:**

There was a significant difference in the prevalence of *A. vasorum* in slugs between cities: 29.4 % in Swansea and 0.3 % in Bristol. In Swansea, prevalence was higher in suburban than in rural and urban areas. Comparing the sampled slug fauna, *Arion rufus* was found in greater numbers in Swansea than Bristol, and was commonly infected (prevalence 41 %). This, alongside the timing of slug collections in summer rather than autumn, could explain low infection prevalence in the Bristol sample. In the absence of *Ar. rufus* as a preferred host for *A. vasorum*, *Ar. flagellus* and *Limacus maculatus* appear to act as versatile hosts that are present in suburban and urban areas in Swansea (prevalence in *Ar. flagellus* 33 %; in *L. maculatus* 44 %) and in Bristol (prevalence in *Ar. flagellus* 0.9 %). These slug species might provide *A. vasorum* with an alternative vehicle to reach the final host, when the main host *Ar. rufus* is scarce or absent.

**Conclusion:**

We conclude that the composition of the slug fauna varies spatially, and that this could help explain patchiness in the prevalence of *A. vasorum*. A suburban peak was found in the prevalence of infection in intermediate hosts, perhaps explained by a higher density of competent intermediate and/or definitive hosts.

## Background

*Angiostrongylus vasorum* is an emerging parasite in dogs, with frequent new reports throughout Europe and beyond [1, 2]. Within several endemic Europe countries, its distribution appears to be expanding, but in a highly heterogeneous or patchy manner. In the United Kingdom (UK), *A. vasorum* was first reported in Cornwall in southwest England in the 1970s [3, 4], later in Wales and southeast England [5–7], and recently in central and northern England and Scotland [8–12]. National questionnaire surveys of veterinary practices confirmed both northward spread and persistent heterogeneity in disease incidence in dogs [13]. Better understanding of the factors underlying this patchy distribution is needed to guide risk assessments by veterinary clinicians and other stakeholders, in order to prevent and control the severe disease that can ensue from infection [14], as well as to build on currently sparse fundamental understanding of the epidemiology of nematode parasites in gastropod intermediate hosts [15, 16].

*Angiostrongylus vasorum* has an indirect life-cycle with canids such as domestic dogs (*Canis lupus familiaris*) and red foxes (*Vulpes vulpes*) as the definitive host, terrestrial gastropod molluscs (slug or snail) acting as intermediate hosts, and frogs acting as paratenic or intermediate hosts [17]. Heterogeneity in parasite abundance and disease risk to definitive hosts could arise from variable environmental influences on mollusc populations and infection rates. Using a recently developed real time polymerase chain reaction (PCR) assay [18], surveys of prevalence in slugs are feasible. Previous studies assessing the presence of *A. vasorum* in slugs and snails have reported variable prevalence. In Denmark, 29 % of molluscs sampled contained larvae and prevalence varied by locality [19]. The species found to be infected in this study were *Arion lusitanicus*, *Ar. ater*, *Ar. aterrufus* and *Limax maximus*. A survey in south Wales (UK) reported 43 % prevalence using PCR, with *Ar. ater* aggregate, *Ar. hortensis* aggregate and *Deroceras* sp. positive [18]. In London (UK), prevalence of larval recovery from slugs was 1.6 %, with a broad host range including two species previously not recorded as acting as intermediate hosts for *A. vasorum*: *Ar.distinctus* and *Tandonia sowerbyi* [20]. In a PCR survey of slugs in west Scotland, an area newly colonised by *A. vasorum*, the slug fauna and positive slugs were dominated by large *Arion* species [11], with overall prevalence of 7 %.

The aims of the present study were to determine the prevalence of infection in slugs in contrasting areas within a region endemic for *A. vasorum*, and to determine whether differences in slug species composition could explain differences in overall prevalence. This information is needed to better understand the role of slugs as intermediate hosts for *A. vasorum*, the factors underlying local risks of transmission, and the most important slug species involved as sources of infection for dogs.

### Ethical approval

Ethical review details are not provided since no formal ethical approval was necessary: the work involves neither work on vertebrates nor human participants or personal data.

## Methods

### Study area

**Fig. 1:**
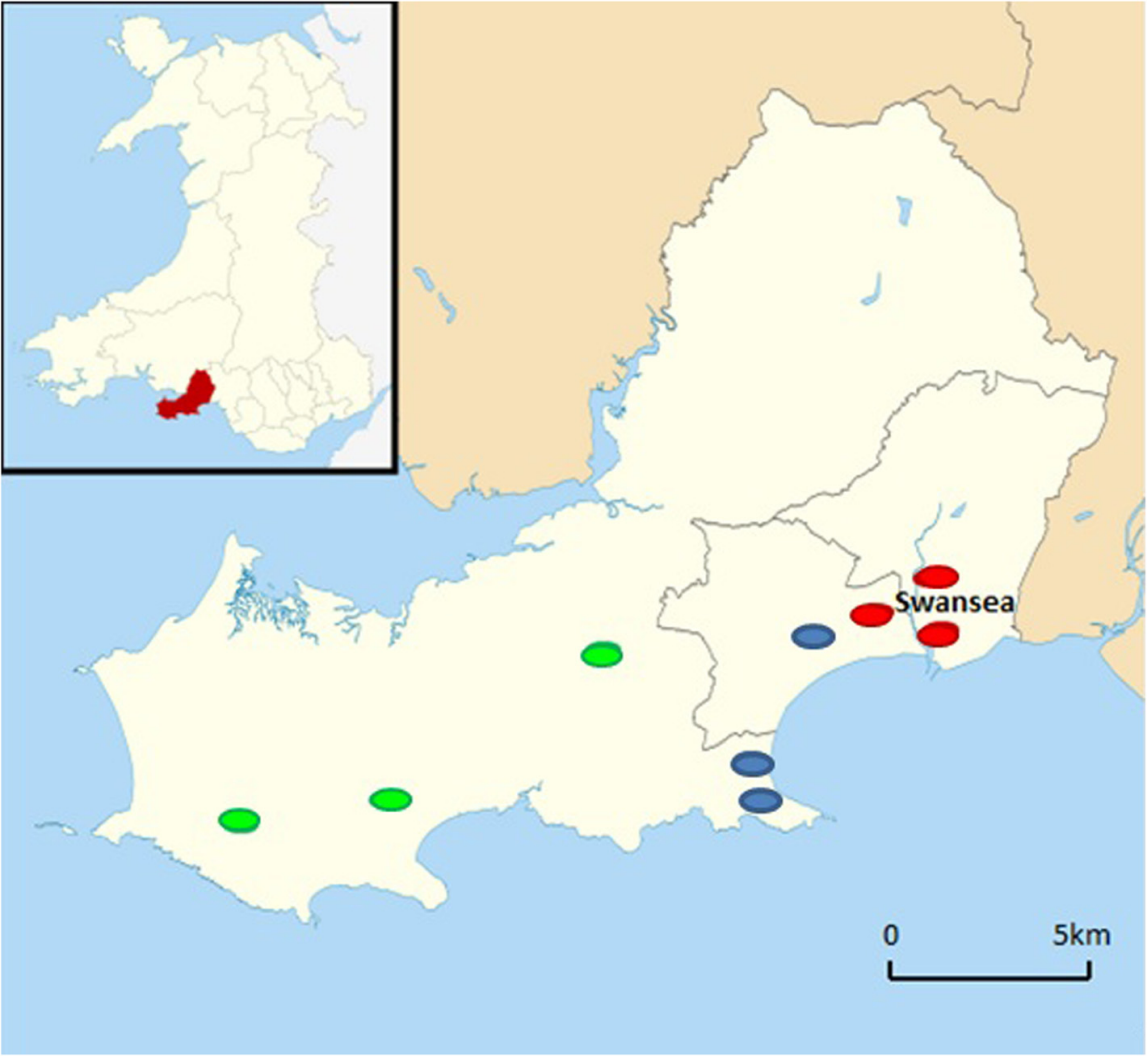
Map of sampling locations in Swansea. Inset: outline and position within Wales of the City and County of Swansea. Sampling sites are marked as follows: red, urban; blue, suburban; green, rural)

**Fig. 2:**
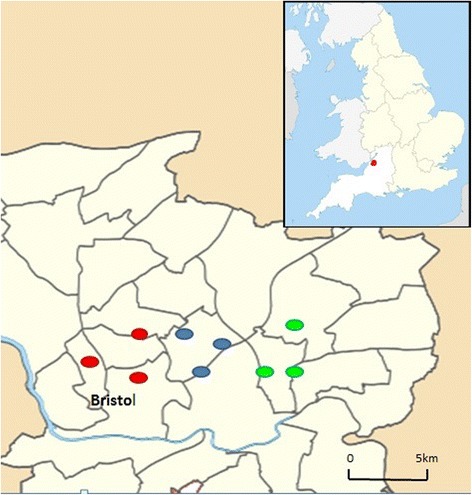
Map of sampling locations in Bristol (inset: location in UK; lines: electoral ward boundaries). Sampling sites are marked as follows: red, urban; blue, suburban; green, rural)

Slugs were collected from parks within a 20 km radius of the centres of two nearby cities, Swansea in south Wales (a known endemic hotspot for *A. vasorum*) (Fig. 1) and Bristol in south-west England (Fig. 2). In Swansea, 180 slugs were collected from October to November 2012, from publicly accessible areas along a rural–urban transect (Table 1). In Bristol, 338 slugs were collected from public parks in July and August 2014 (Table 2). In each location, nine sites were sampled across three broad habitat types (urban, suburban and rural), which were categorised subjectively. Urban areas were close to the city centres, and either built-up (e.g. housing, or tarmacadam-based landscaping) or brown-field (i.e. unused and unmanaged previously built-up land). Suburban areas were composed of a mixture of housing with private gardens, and publicly accessible green space (i.e. public parks), from which slugs were sampled. Rural areas were publicly accessible amenity areas outside the city boundaries, comprising open green fields and woodland, with sampling undertaken from woodland-grassland and ecotones.

**Table 1:** Locations sampled in Swansea in autumn 2012

Site	Abbreviation	Description	Number of slugs	Date of collection
Underhill park	UP	Suburban	20	13.x.2012
Cwmdonkin park	CP	Suburban	20	14.x.2012
West cross	WC	Suburban	20	18.x.2012
Woodlands terrace	WT	Urban	20	21.x.2012
Sainsbury’s car park	SC	Urban	20	23.x.2012
Landore	L	Urban	20	25.x.2012
Penrice	P	Rural	20	29.x.2012
GelliHir	GH	Rural	20	30.x.2012
Pilton green	PG	Rural	20	01.xi.2012

**Table 2:** Locations sampled in Bristol in summer 2014

Site	Abbreviation	Description	Number of slugs	Date of collection
St Andrews park	SAP	Urban	23	05.vii.2014
St George park	SGP	Urban	19	10.vii.2014
Oldbury Court Estate	OCE	Urban	50	14.vii.2014
Warmley forest park	RWH	Suburban	44	16.vii.2014
Overscourt wood	WFP	Suburban	73	19.vii.2014
Rodway Hill	OCW	Suburban	28	20.vii.2014
Wapley Bushes nature reserve	WBN	Rural	47	25.vii.2014
Golden Valley nature reserve	GVN	Rural	26	23.vii.2014
Brockwell park	BWP	Rural	28	02.viii.2014

### Collection and processing of slugs

Slugs were collected by actively searching the ground and surface vegetation, soon after dusk by torchlight. All slugs were stored together in labelled plastic boxes according to sampling site. In Swansea, immediate examination of slugs was not possible and so slugs were frozen at -80 °C until used for DNA extraction. Slugs collected in Bristol were kept at 15 °C and processed within a few days of collection: boxes were lined with moistened tissue paper and supplemented with lettuce and carrot as a food source. Individual slugs were identified using Rowson et al. [21, 22] and were assigned either to genus or species level. Following identification, slugs were killed by decapitation, and 10 mg of foot tissue used for DNA extraction and real time PCR assays. Very few snails were observed and none were collected.

### DNA extraction and real time PCR assays

DNA was extracted using DNEasy Blood and Tissue Kit (Qiagen, Germany) according to the animal tissue extraction protocol, with final elution volume of 100 μl. A portion of the ITS-2 (internal transcribed spacer 2) gene (180 base pairs) was amplified using the following primers: I2F2 5′-GCGTGTGTTCATGTTTGGAC-3′and I2R2 5′-CATTACTAGCATACAAGCACATG-3′ [18]. Real-time PCR assays were performed in a final reaction volume of 25 μl that consisted of 12.5 μl of 2× Quantifast SYBR Green PCR Master Mix (QIAGEN, Germany), 1 μM of each primer and 6.5 μl of dH_2_O. Cycling conditions involved an initial activation step of 95 °C for 5 min followed by 40 cycles of 95 °C for 10 s and 62 °C for 30 s using a Mx3005P quantitative PCR system (Stratagene, California, USA). For all assays, positive (adult *A. vasorum* DNA) and negative (absence of DNA template) controls were included.

For samples from freshly collected slugs, higher fluorescence on 10-fold dilution of some extractions suggested the possible presence of PCR inhibitors as seen in previous studies [18]. All extractions were diluted 1:10 to surmount this. The cycle number was increased from 40 to 50 following dilution to allow sufficient amplification of PCR product.

In order to check for false negatives arising from localisation of larvae outside the 10 mg foot muscle sample, a total of 58 slugs testing negative by PCR, from all localities of Swansea [suburban (*n* = 16); urban (*n* = 21) and rural (*n* = 21)] were homogenised. The slugs were individually homogenised using a mortar and pestle and 25 mg of tissue randomly taken for DNA extraction and real time PCR analysis.

### Statistical analysis

The prevalence of *A. vasorum* was compared across localities using Chi-square tests, and across slug species using Fisher’s exact test, in both cases using global prevalence to calculate expected frequencies. To describe variation in slug species composition in Bristol and Swansea, Principal Components Analysis (PCA) was used, with sample site set as group and slug species as the list of variables (SPSS Statistics version 21, IBM, USA). Chi-square tests were further used to compare the relative frequency of individual slug species in samples from different localities. *P* values of < 0.05 were considered to be statistically significant.

## Results

### Prevalence of *Angiostrongylus vasorum* in slugs

**Fig. 3:**
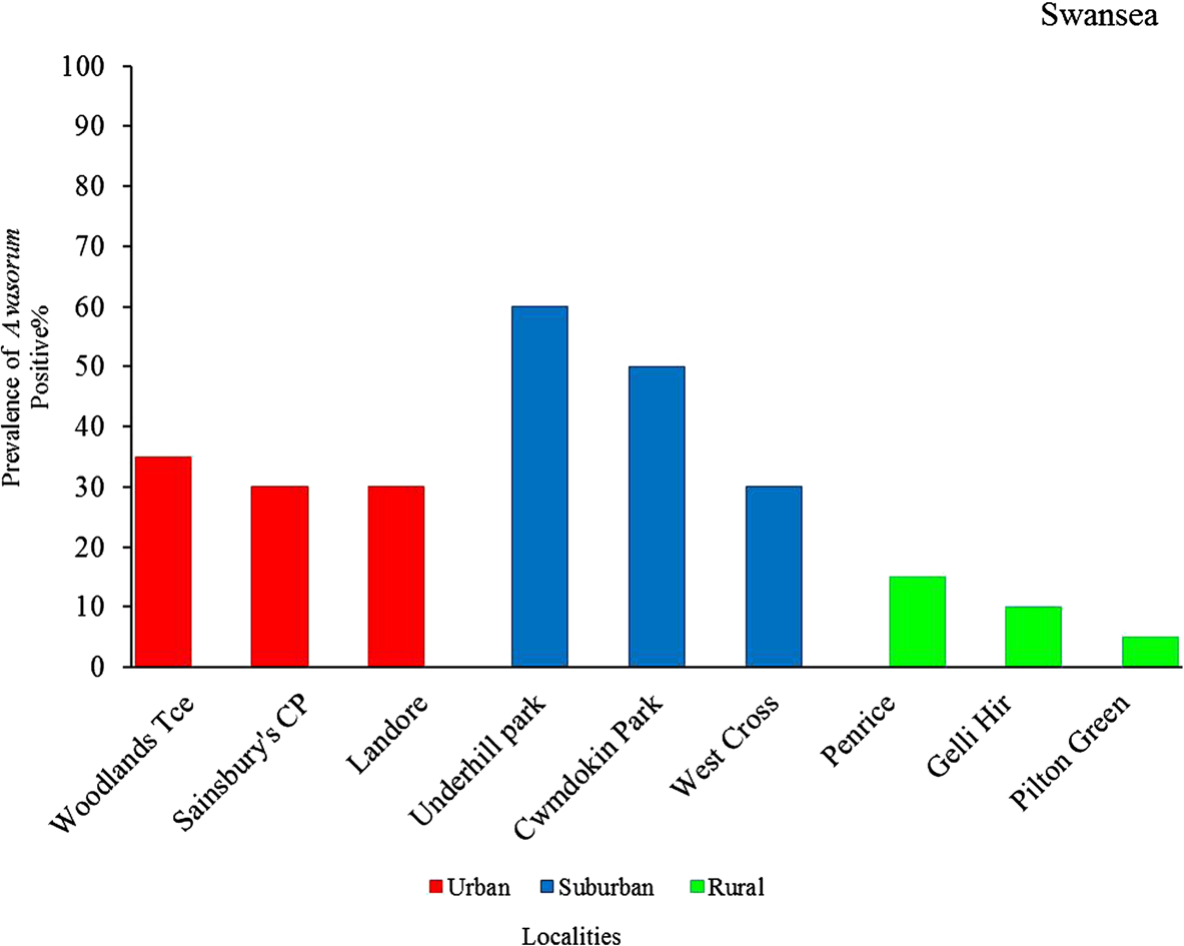
Prevalence of slugs infected with *Angiostrongylus vasorum* as determined by real-time PCR, across nine locations spanning the rural–urban gradient in Swansea

In Swansea, of the 180 slugs examined for *A. vasorum* infection, 53 (29.4 %) were positive by real time PCR assay. More than half of all infected slugs were recorded in suburban environments (Fig. 3). The prevalence of *A. vasorum* was significantly different between the habitats sampled (urban, suburban and rural; *χ*^2^ = 14.78, d.f. = 2, *p* < 0.001). There was no significant difference in prevalence between suburban and urban habitats (*p* = 0.15); however, infection was significantly more common in suburban than in rural slugs (*χ*^2^ = 14.16, d.f. = 1, *p* < 0.001). There was also substantially higher prevalence in urban areas compared to rural areas (*χ*^2^ = 14.54, d.f. = 1, *p* < 0.001). In Bristol, only one of the 338 slugs tested positive for *A. vasorum*, and this was collected from a suburban location (Table 3), giving a prevalence of 1.3 % for that park and a prevalence of 0.3 % overall for the sites in Bristol. All slugs homogenised (*n* = 58) for identification of false negatives gave a negative result.

**Table 3:** Composition of gastropod collections made in Swansea in 2012 and Bristol in 2014, and prevalence of *Angiostrongylus vasorum* using real-time PCR. *N* = number of each species collected, and number and percentage positive by PCR

Gastropod I.D.			Location of collection					
Family	Genus	Species	Swansea			Bristol		
			*N*	*N* positive	% positive	*N*	*N* positive	% positive
Arionidae	*Arion*	*rufus*	68	28	41	27	0	0
	*Arion*	*flagellus*	27	7	26	115	1	0.9
	*Arion*	*subfuscus*	1	0	0	5	0	0
	*Arion*	*distinctus*	–	–	–	3	0	0
	*Arion*	*owenii*	–	–	–	12	0	0
	*Arion*	*ater*	–	–	–	16	0	0
	*Arion*	*fasciatus*	–	–	–	3	0	0
Agriolimacidae	*Deroceras*	*reticulatum*	3	0	0	93	0	0
Limacidae	*Limax*	*maximus*	1	0	0	12	0	0
	*Limacus*	*maculatus*	18	8	44	30	0	0
Milacidae	*Tandonia*	*sowerbyi*	4	0	0	22	0	0
Total			122	43	35.2	338	1	0.3

### Comparison of slug species across locations

**Fig. 4:**
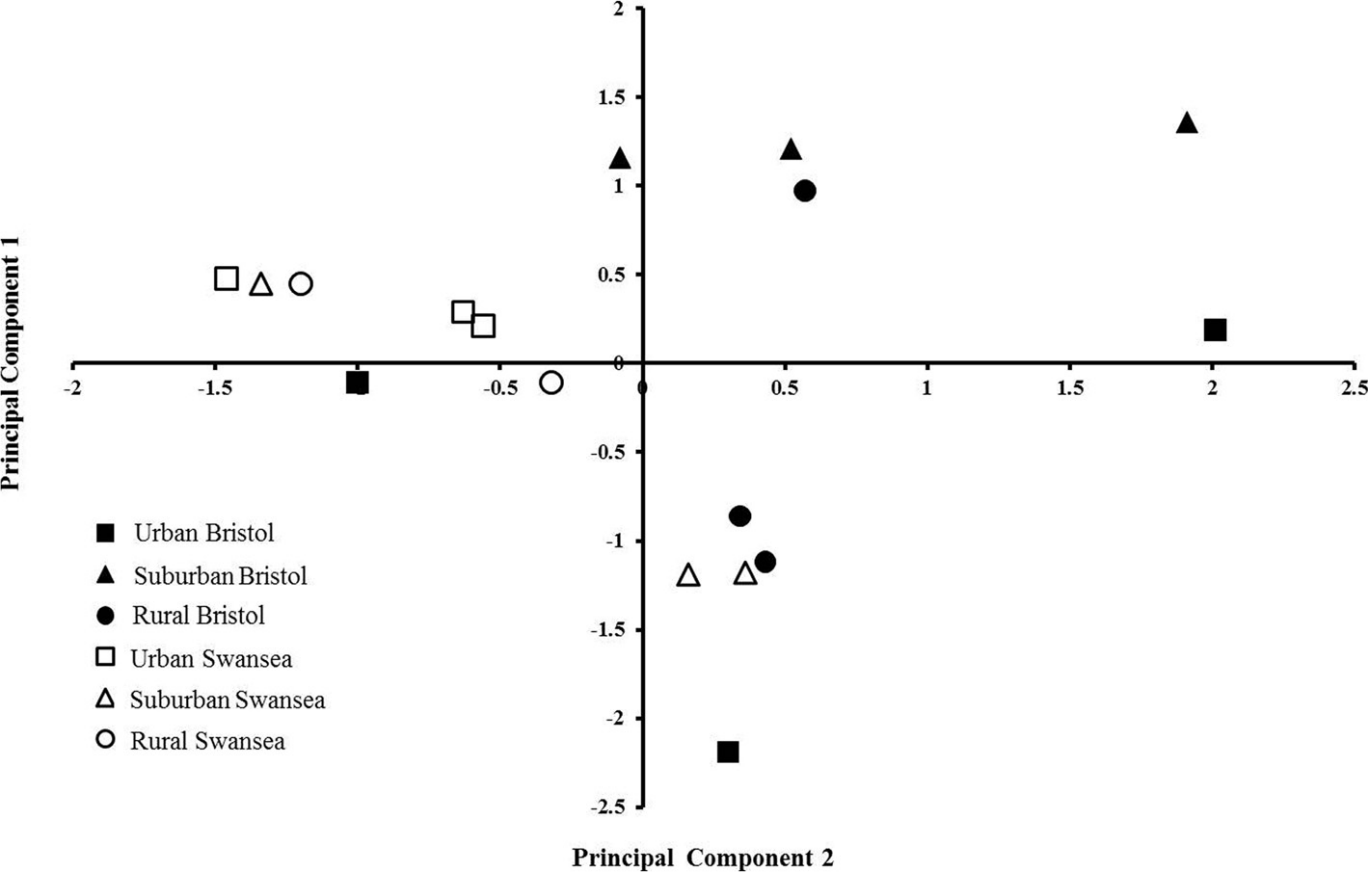
Ordination of sample location on the first two principal component axes of the Principal Components Analysis (PCA). See Table 4 for component loadings and eigenvalues

Principal components analysis (PCA) revealed differences in the composition of the slug fauna in different locations (Table 4), but no clear overall pattern along the urban-rural gradient or between the two cities (Fig. 4). Using univariate Chi-square tests, however, *Ar. rufus* was found to be more common in all habitats sampled in Swansea compared with Bristol (Table 5). By contrast, *Ar. flagellus* was more common at sub-urban and rural habitats in Bristol than in Swansea. *Deroceras reticulatum* and *L. maximus* were also more common in Bristol than in Swansea. *Arion flagellus* was significantly more common at combined suburban and urban than rural habitats in Bristol (Table 6), while *Ar. owenii* and *T. sowerbyi* were more common in rural locations. In Swansea, *L. maculatus* was found only in the urban habitats.

**Table 4:** Component loadings for the first two principal components (PC), used to compare slug species composition between sampled locations. PC1 (eigenvalue 1.68) explained 24 % of the total variance; and PC2 (eigenvalue 1.59) explained 23 % of the total variance

Variable (slug species)	Component 1	Component 2
*A. rufus*	0.50	-0.77
*A. flagellus*	0.64	0.55
*A. ater*	0.45	0.01
*D. reticulatum*	0.20	0.78
*L. maximus*	-0.10	-0.23
*L. maculatus*	-0.85	0.17
*T. sowerbyi*	-0.23	0.06

**Table 5:** Number of specimens of each slug species collected in Swansea and Bristol

Species	Urban		Suburban		Rural	
	Swansea	Bristol	Swansea	Bristol	Swansea	Bristol
*A. rufus*	16^a^	7^a^	33^b^	15^b^	19^c^	5^c^
*A. flagellus*	5	6	13^b^	92^b^	9^c^	17^c^
*A.subfuscus*	1	0	0	4	0	1
*A.distinctus*	0	3	0	0	0	0
*A. owenii*	0	12	0	0	0	0
*A. ater*	0	3	0	8	0	5
*A. fasciatus*	0	0	0	3	0	0
*D. reticulatum*	3^a^	44^a^	0^b^	25^b^	0^b^	24^b^
*L. maximus*	0^a^	10^a^	0	0	1	2
*L. maculatus*	18	21	0	0	0	9
*T. sowerbyi*	0	1	0	1	4	20
Total	43	92	46	148	33	98

Super-scripts show significant differences (*p* < 0.05) using Chi-square when comparing: ^a^urban localities in Swansea and Bristol; ^b^suburban localities in Swansea and Bristol; ^c^ rural localities in Swansea and Bristol

**Table 6:** Number of specimens of each slug species collected in Swansea and Bristol, pooling urban and suburban environments in each area

Species	Swansea		Bristol	
	Urban and suburban	Rural	Urban and suburban	Rural
*A. rufus*	49	19	22	5
*A. flagellus*	18	9	98^b^	17^b^
*A. subfuscus*	1	0	4	1
*A. distinctus*	0	0	0	3
*A. owenii*	0	0	0^b^	12^b^
*A. ater*	0	0	11	5
*A. fasciatus*	0	0	3	0
*D. reticulatum*	3	0	69	24
*L. maximus*	0	1	10	2
*L. maculatus*	18^a^	0^a^	21	9
*T. sowerbyi*	0	4	2^b^	20^b^
Total	89	33	240	98

Super-scripts show significant differences (*p* < 0.05) using Chi-square when comparing: ^a^(sub-)urban and rural localities in Swansea; ^b^(sub-)urban and rural localities in Bristol

## Discussion

These results demonstrate for the first time a rural–urban gradient in *A. vasorum* prevalence in intermediate hosts in the UK. Higher prevalence of *A. vasorum* in suburban areas is consistent with studies in Denmark in gastropods [19] and in foxes [23]. Peak prevalence in suburban areas could be explained by overlap of high fox population density and high density of suitable intermediate hosts, as proposed for the fox tapeworm, *Echinococcus multilocularis*, in Europe [24].

Wide variation in the prevalence of *A. vasorum* at different sites and between the cities of Swansea and Bristol demonstrates a high level of spatial heterogeneity in parasite distribution among intermediate host populations. Previous studies have noted marked geographical patchiness of angiostrongylosis in definitive hosts [25–28]. The reasons for this patchy distribution are unknown [30], and could include spatial variation in intermediate host density and species composition, and in environmental factors influencing transmission, as well as local movement of infected intermediate hosts around new parasite introductions [30–32].

The prevalence of 29.4 % in Swansea in the current study is considerably higher than the 1.6 % recorded by Patel et al. [20] in London, by extraction and identification of larvae from gastropod tissues. Larval extraction methods are likely to underestimate true infection prevalence, through low sensitivity and because pre-infective stage larvae are usually not recovered [Abdul-Aziz, unpublished observations]. Conversely, prevalence estimated from PCR does not accurately reflect the presence of mature larvae able to propagate infection in definitive hosts, since earlier larval stages and, potentially, residual DNA from non-viable larvae can also be detected [11]. Prevalence estimates using these different methods are, therefore, not comparable. Low prevalence in a large sample size in the present study in Bristol suggests that false positive results are not common using this PCR assay.

In the present study, slug species were identified by external and internal morphology based on Rowson et al. [22], a new comprehensive guide to British and Irish slugs validated by PCR amplification and sequencing of mitochondrial (16S, COI) and nuclear (ITS-1) DNA. Previous work on *A. vasorum* in slugs [18–20] examined ‘aggregates’ of superficially similar species. The present study therefore provides more accurate information on species distribution of *A. vasorum* based on more specific identification.

The present study found broadly similar slug species composition in the nearby cities of Swansea and Bristol. Together with the wide host range observed here and in previous studies, this suggests that the overall prevalence of *A. vasorum* is not closely related to the presence or absence of particular intermediate host species. Nevertheless, some differences in slug fauna were noted. Sampling in Swansea yielded fewer species overall, even allowing for lower sample size, and less variability in slug species between sites compared with Bristol. *Arion rufus* was relatively more common across locations in Swansea, and *Ar. flagellus* and some of the smaller slug species were more common in Bristol. Reasons for these differences could include ecological factors (e.g. climate, soil, habitat, or predation) or a history of accidental introduction (perhaps especially for those species found at single sites). However, the consistent differences in abundance of widespread species present at all sites (*Ar. rufus* and *Ar. flagellus*) seem more likely to be due to ecological rather than historical factors. *Arion rufus*, widely found and commonly infected in the Swansea samples, has been acknowledged to act as an efficient host of *A. vasorum* and to be a major potential source of infection in various locations [18, 33, 34]. It appears that in urban areas of Swansea, *L. maculatus* acts as additional or secondary host. In Bristol, the lower availability of *Ar. rufus* as the preferred or optimal host of *A. vasorum* in Bristol might explain the low overall infection prevalence. Nevertheless, *Ar. flagellus* and *L. maculatus* were abundant and could be important alternative hosts in such areas.

The broad intermediate host range observed in this study is in agreement with previous data on experimental and natural infections [18–20, 35]. Around 25 species have been successfully infected in an experimental setting. These are: (i) terrestrial slugs (*Deroceras reticulatum, D. agreste, D. laeve, Arion lusitanicus, Ar. hortensis, Limax flavus, Laevicaulus alte*); (ii) terrestrial snails (*Arianta arburstorum, Bradybaena similaris, Cochlodina laminata, Cepea nemoralis, Euparypha pisana, Prosopeas javanicum, Helix aspersa, Helix pomatia, Subulina octona, Succinea putris, Achatina fulica,Vitrea diaphana*); (iii) aquatic snails (*Lymnaea peregra peregra, Lymnaea tomentosa, Planorbis planorbis, Biomphalaria glabrata, Anisus leucostomus, Physa* sp.) [33, 35–40]. In natural infections, mollusc species reported to be hosts for *A. vasorum* were: Arionidae: *Arion ater* aggregate (including *A. ater* seg.*, A. rufus* seg.*, A. lusitanicus* auctt.), *Arion hortensis* aggregate (including *A. distinctus*), *Arion subfuscus* aggregate (including *A. subfuscus* seg.), *Geomalacus maculatus*; Agriolimacidae: *Deroceras* sp.*, Deroceras reticulatum, Deroceras caruanae* auctt.*, Deroceras laeve,* Limacidae: *Limax* sp.*, Limax marginatus, Limax maximus, Limacus maculatus*; Milacidae: *Milax* sp.*, Tandonia sowerbyi*; Snails: *Cornu aspersum* [4, 11, 18–20, 33–35, 38, 41]. However, the extent to which these types of mollusc are accessible to definitive hosts may vary. A broad intermediate host range has also been reported for other *Angiostrongylus* species, such as *A. cantonensis* [42, 43] and *A. costaricensis* [44, 45].

The slugs most commonly infected in this study were larger *Arion* spp., which have been previously found to have higher larval burdens than smaller slugs [41, 46]. Rosen et al. [38] reported average infection levels in *Ar. rufus* of 750 L3 and over 1000 in some cases. *Arion flagellus* and *L. maculatus*, alternative hosts for *A. vasorum* in the present study, are also among the larger slug species. It is possible that these larger slugs are more commonly infected than others through greater coprophagic tendencies. *Arion flagellus* was found to be a new host record for *A. vasorum*. There is evidence of ongoing distributional spread of both *Ar. flagellus* and *L. maculatus* in the UK and elsewhere [21, 22].

The current study demonstrated infection of slugs with *A. vasorum* in summer and autumn, confirming previous records in summer [18], autumn [19] and summer and winter [20]. Thus, slugs are likely to present a source of infection to dogs all year round. The much lower prevalence in Bristol in summer, compared with that in Swansea in autumn, might stem from a seasonal rather than a spatial effect, and results are therefore not comparable between locations. Higher prevalence in autumn might be expected based on accumulation of infection and maturation of larvae through the summer months. More information is needed on seasonal variation in *A. vasorum* prevalence in intermediate hosts, to contribute to better understanding of the seasonal epidemiology and risks of infection to dogs, and also to guide efficient sampling for surveillance or spatial studies.

## Conclusions

In conclusion, the results presented here demonstrate heterogeneity in the distribution of *A. vasorum* infection in slugs in two nearby cities, and variation in prevalence along the rural-urban gradient. The composition of the slug fauna appears to play a role in parasite distribution at these scales. Further studies are needed to account for ecological and host factors underlying species differences in intermediate host infections, and to separate spatial from seasonal drivers of prevalence.
